# Technological, Functional, and Sensory Evaluation of Sorghum Extruded Snacks Enriched with Chokeberry Pomace

**DOI:** 10.3390/foods15111959

**Published:** 2026-06-02

**Authors:** Elizabet Janić Hajnal, Lato Pezo, Vojislav Banjac, Dubravka Škrobot, Vanja Travičić, Bojana Filipčev, Bojana Perduh, Jovana Kos, Olivera Šimurina, Biljana Cvetković

**Affiliations:** 1 Institute of Food Technology, University of Novi Sad, Bulevar cara Lazara 1, 21000 Novi Sad, Serbia; vojislav.banjac@fins.uns.ac.rs (V.B.); dubravka.skrobot@fins.uns.ac.rs (D.Š.); bojana.filipcev@fins.uns.ac.rs (B.F.); bojana.radic@fins.uns.ac.rs (B.P.); jovana.kos@fins.uns.ac.rs (J.K.); olivera.simurina@fins.uns.ac.rs (O.Š.); biljana.cvetkovic@fins.uns.ac.rs (B.C.); 2 Institute of General and Physical Chemistry, University of Belgrade, Studentski Trg 12-16, 11000 Belgrade, Serbia; latopezo@yahoo.co.uk; 3 Faculty of Technology, University of Novi Sad, Bulevar cara Lazara 1, 21000 Novi Sad, Serbia; vanjaseregelj@tf.uns.ac.rs

**Keywords:** chokeberry pomace, extrusion, functional foods, phenolic compounds, response surface methodology, artificial neural network

## Abstract

This study investigated the effect of enriching extruded sorghum-based snacks with chokeberry (*Aronia melanocarpa*) pomace on their nutritional, functional, and sensory properties. The formulations varied from 100% sorghum (the control) to blends with up to 30% pomace, with selected samples supplemented with 1% cinnamon. Increasing the pomace content was accompanied by a marked rise in total monomeric anthocyanins (from not detected in the control to 25.12 mg/100 g at 30% pomace) and significantly enhanced antioxidant activity. *Free DPPH* values increased from 3.45 to 18.65 mmol TE/100 g, while *Free ABTS* values rose from 3.12 to 25.98 mmol TE/100 g. The highest antioxidant capacity was observed in samples containing 27–30% pomace. The incorporation of cinnamon (1%) further improved antioxidant activity compared to corresponding formulations without cinnamon (e.g., *Free DPPH* up to 10.79 mmol TE/100 g at 13% pomace). Higher pomace levels also produced increased crispiness and brittleness due to increased insoluble fiber content. Response surface methodology and artificial neural networks confirmed strong links among the formulation, processing conditions, and product quality. Sensory evaluation revealed overall liking scores below the neutral level (5 of the 9 hedonic scales), with bitterness identified as the main limitation, despite favorable texture attributes. Correspondence analysis of consumer feedback suggested potential strategies for sample improvement, including bitterness reduction and flavor enhancement, through the addition of sweet, salty, or complementary flavor notes (e.g., chocolate, nutty, fruity, and warm spices). Overall, chokeberry pomace shows promise as a functional ingredient in sorghum snacks, although further sensory optimization is required to enhance consumer acceptance.

## 1. Introduction

Sorghum (*Sorghum bicolor* (L.) Moench) is the fifth major cereal globally, with world production reaching approximately 60 million tons annually, primarily from the USA, Nigeria, and India [1,2]. Utilization patterns vary significantly by region. In the period 2015–2024, the production share of sorghum grain by region was the following: Africa (47%), USA (35%), Asia (14%), Oceania (3%), and Europe (2%) [3]. Its utilization varies significantly by region. In Africa and parts of Asia, sorghum serves as a staple food crop, particularly for low-income populations, and is processed into traditional foods such as rotis, porridges, and beverages [4,5]. However, consumption as a food staple has declined in Asia due to rising incomes, urbanization, and government policies favoring rice and wheat [5]. In developed countries like the USA, Australia, and China, sorghum is primarily used for livestock feed and fodder [2]. Despite its nutritional quality and stress tolerance, sorghum faces challenges including processing difficulties and the limited availability of ready-made products [4]. Therefore, the development of innovative sorghum-based products represents an important opportunity to increase its utilization in human nutrition. Devi et al. [6] reported that enrichment techniques include protein fortification, micronutrient enhancement, and advanced extrusion processing. Protein enrichment strategies involve adding whey protein isolate, soy flour, or legume flour, which can increase the protein content to 19–21% and improve overall nutritional quality [7]. Genetic approaches have also been developed to enhance pro-vitamin A, iron, and zinc bioavailability, potentially providing 20–90% of nutritional requirements [8]. Extrusion processing plays a crucial role, with studies showing that carefully controlled temperature and moisture can optimize the expansion ratio, protein digestibility, and sensory attributes [9]. In contrast, the use of fruit and vegetable by-products, particularly pomace, in sorghum-based snacks remains limited. Fruit-based enrichment has been demonstrated with baobab fruit powder, where sorghum snack bars supplemented with sesame and baobab fruit pulp powder showed improved nutritional profiles, with protein contents ranging from 11.28–16.74 g/100 g and enhanced mineral contents, including iron (5.46–14.61 mg/100 g) and calcium (82–246 mg/100 g) [10]. On the other hand, vegetable enrichment research includes pumpkin pulp and seed incorporation, where fermented sorghum flour blended with pumpkin components achieved significant nutritional improvements, with the highest blend (60:20:20 ratio) reaching 22.87% protein content and 875.00 µg RAE/100 g vitamin A [11]. However, the research primarily focuses on protein enrichment through legume flours, soy, and whey proteins rather than fruit/vegetable pomace specifically [6]. These studies do not specifically address pomace, which is a rich source of dietary fiber and bioactive compounds. Therefore, a clear research gap exists regarding the application of fruit and vegetable pomace in extruded sorghum snacks and its impact on product quality. Chokeberry (*Aronia melanocarpa*) pomace can enhance the nutritional profile of snacks, provide a rich source of bioactive compounds and dietary, while maintaining desirable sensory qualities. Incorporating chokeberry pomace into cereal snacks not only boosts their nutritional value but also aligns with consumer preferences for functional foods with enhanced nutritional and antioxidant potential [12,13]. Although chokeberry pomace has shown potential in baked and freeze-dried products [13,14], its application in sorghum extrusion remains unexplored, particularly regarding the effect of high insoluble fiber content on extrusion process parameters and anthocyanin retention. Moreover, its utilization supports sustainable food production through by-product valorization and aligns with current consumer demand for functional foods [12,14].

Therefore, this study aims to develop and evaluate sorghum-based extruded snacks enriched with chokeberry pomace, with particular emphasis on optimizing technological, functional, and sensory properties using both response surface methodology (RSM) and artificial neural networks (ANNs).

## 2. Materials and Methods

### 2.1. Material Grinding and Mixing

A hammer mill (model 9FQ-50, XT Machinery, Zouping, China) equipped with a 1 mm diameter sieve was utilized to finely grind approximately 100 kg of commercially acquired white sorghum grain (*Sorghum bicolor* (L.) Moench) purchased from Farm Commerc d.o.o. (Čantavir, Serbia). Chokeberry pomace, obtained as a by-product of juice production (Zdravo Produkt d.o.o., Ridjica, Serbia), was freeze-dried in an industrial lyophilizer (FD 100, Pigo, Belgrade, Serbia). The chokeberry pomace prior to freeze-drying contained approximately 62.6% moisture. Lyophilization was performed under a vacuum conditions ranging from approximately 0.01 to 0.05 kPa, with the temperature of the evaporator maintained between −45 and −50 °C. The total drying time was 16 h. After lyophilization, the moisture content was reduced to below 10%. The dried pomace was subsequently milled using a cross-beater mill (SK1, Retsch GmbH, Haan, Germany) with a 1 mm sieve to obtain a fine powder. The resulting pomace powder, together with commercially purchased Ceylon cinnamon, was used as a functional ingredient in the formulation of extruded sorghum-based snacks. Commercially available Ceylon cinnamon powder was purchased from a local retail market in Novi Sad, Serbia.

### 2.2. Extrusion Conditions

Sorghum-based snacks were produced using a co-rotating twin-screw extruder (Bühler BTCM-30, Bühler, Uzwil, Switzerland) with total barrel length of 880 mm and a length-to-diameter ratio of 28:1. Various snacks were made by including the ground chokeberry pomace in sorghum flour at levels from 2 to 30%, as well as adding cinnamon at 1% (Table 1). Cinnamon (1%) was incorporated into selected formulations (trials 11–13) to evaluate its potential contribution to flavor improvement and consumer acceptability. Dry mixes of sorghum and chokeberry were prepared by mixing the ingredients in a twin-shaft paddle mixer (Forberg, Norway) for 90 s. The extruder barrel consisted of seven sections and was fitted with two tempering devices, enabling regulation of the water temperature to either heat or cool specific barrel sections. Sections 2–4 of the extruder barrel were maintained at 80 °C using the first tempering tool, while Sections 6 and 7 of the extruder barrel were held at 120 °C by the second. A die plate with a conical inlet and die length of 10.1 mm and a single aperture of 4 mm in diameter was employed, corresponding to a total die area of 12.56 mm^2^. The screw configuration applied was specifically designed for the production of directly expanded products [15]. The feed rate of dry materials was set at 50 kg/h, and the screw speed was set at 900 RPM and kept constant for the production of all samples. To ensure constant moisture levels of the material in the barrel (approximately 15%), water was injected directly via a cavity pump in Section 2 of the barrel. At the die head, sensors continuously monitored both the temperature and pressure of the extruded material.

All extrusion parameters, including the die temperature (*T*), die pressure (*P*), motor load (*Torque*), and specific mechanical energy (*SME*), were accessible in real time through the extruder’s control screen. The output cutter, equipped with six rotating knives operating at 700 RPM, ensured the desired product length. After running the extruder under steady conditions for at least two minutes, approximately 0.5 kg of product was collected on four occasions at one-minute intervals to obtain a representative sample. Following extrusion, products were cooled at ambient temperature, packaged in plastic bags, and subsequently prepared for further analysis.

### 2.3. Determination of Physicochemical Properties of Snacks

The bulk density (*BD*) of the sorghum snack was measured in triplicate using a bulk density tester (Tonindustrie, West & Goslar, Germany).

The expansion ratio (*ER*) was determined by measuring the cross-sectional diameter of the sorghum-based extruded snacks with a sliding caliper with a Vernier scale. The ratio was calculated by dividing the cross-sectional diameter of the extrudate by the diameter of the die opening [16,17]. Ten random samples from each trial (0–13) were utilized to calculate the *ER* values.

The water absorption index (*WAI*) and water solubility index (*WSI*) were assessed following the methodology established by Anderson et al. [18], with minor modifications as noted by Janić Hajnal et al. [19]. The results for both *WAI* and *WSI* are presented as mean values calculated from four extruded samples in each study.

The total, soluble, and insoluble dietary fiber in both the raw materials and extruded products was assessed using the established AOAC 991.43 Official Method [20].

### 2.4. Color of Snacks

The color of the sorghum snack (L*, a*, b*) was measured using a Chroma Meter Konica Minolta CR-400 (Minolta, Tokyo, Japan) equipped with a CR-A33 attachment, calibrated with a white standard plate under D65 illumination and a 10° standard observer angle. Six measurements were obtained from the surface of the milled (KnifetecTM 1095 mill, Foss, Hoganas, Sweden) and homogenized sorghum extrudate in each trial. The L* value (0–100) quantifies brightness from black to white; the a* (+/−) value indicates red/green chromaticity; the b* (+/−) value denotes yellow/blue chromaticity; the C* value assesses color intensity and saturation.

### 2.5. Texture Analysis

Chokeberry pomace-enriched sorghum extruded snacks were analyzed for textural properties by performing a uniaxial “bulk” compression test as described in Janić Hajnal et al. [21]. A compression test was performed using an Ottawa cell operated with a 17-bladed extrusion plate on a texture analyzer model TA-XTplus (Stable Micro Systems, Godalming, Surrey, UK). Sorghum-based extruded snack samples were arranged in a single layer and compressed at a 5 mm/s test speed and probe distance of 57 mm. The compression test was performed in 20 repetitions. The mechanical properties (hardness and crispness) of the sorghum-based extruded snacks were assessed by analyzing the obtained multi-peak force–time curve using the algorithm of Exponent software v. 6.1.18.0. Hardness (*Hard*) was extracted from the force–time curves as the maximum peak during the test. Further analysis of the multi-peak compression curves included calculations of the following parameters: crispness by number of fractures (*NoFract*), area under curve, normalized curve length and mean compression force. These parameters were used to calculate the crispiness work (*Crisp work*) and index (*Crisp index*) as indicated in the studies by Van Hecke et al. [22] and Heidenreich et al. [23], according to the following equations:

*Crisp work* = *A*/*N* (1)

*Crisp index* = *L_N_*/*A* × *F_mean_* (2)

where *Crisp work* (N mm) is the crispiness work; *Crisp index* (×10^−3^) is the crispiness index; *N* is the number of peaks; *L_N_* is the normalized curve length (mm/N), calculated as the ratio of the (length of the actual curve (mm) and maximum force (N)); *A* is the area under the compression curve (N mm); and *F_mean_* (N) is the sum of the actual force values divided by the number of peaks. A higher *Crisp index* combined with a lower *Crisp work* value is indicative of a crispy texture.

### 2.6. Anthocyanin Content

Prior to analysis, anthocyanins were extracted from homogenized extruded snack samples and chokeberry pomace using an acidified ethanol/water solution. Briefly, 1.00 g of dried and homogenized sample was mixed with 20 mL of ethanol/water/HCl solution (80:19:1, *v*/*v*/*v*) and extracted under light-protected conditions for 60 min at room temperature with continuous shaking. The extracts were centrifuged at 5000× *g* for 10 min, and the supernatants were collected and used for monomeric anthocyanin determination. The monomeric anthocyanin content in the extruded snacks enriched with chokeberry pomace and in the chokeberry pomace samples was quantified using a pH differential method adapted to a 96-well microplate format (Thermo Fisher Scientific Inc., Waltham, MA, USA), following the approach described by Lee et al. [24] with minor modifications. Each of the extruded snacks enriched with chokeberry pomace and the chokeberry pomace sample were analyzed in triplicate. Absorbance values were recorded at different pH conditions to account for the structural transformation of anthocyanins. The final concentrations were calculated based on the molar absorptivity and molecular weight of cyanidin-3-glucoside and expressed as mg cyanidin-3-glucoside equivalents (C3G) per 100 g dry weight of the sample.

### 2.7. Free and Bound Phenolics

#### 2.7.1. Extraction and Determination of Total Phenolic Compounds

Phenolic compounds from the extruded snack samples enriched with chokeberry pomace were extracted using an alkaline extraction procedure, adapted from Gopalan and Nampoothiri [25] with minor modifications. The homogenized sample (1.00 g) was suspended in 50 mL of 2 N NaOH and incubated at 25 °C for 12 h under continuous agitation. After incubation, the mixture was acidified to approximately pH 3.5 using 5 N HCl, followed by liquid–liquid extraction with ethyl acetate (three extraction cycles). The combined organic phases were evaporated under reduced pressure using a rotary evaporator (R210, Büchi, Flawil, Switzerland) until near dryness. The obtained residue was reconstituted to 10 mL with an ethanol/water mixture (80:20, *v*/*v*) and protected from light until further analysis. The applied alkaline extraction procedure may contribute to the partial degradation of some phenolic compounds, particularly anthocyanins; however, it was selected to improve the extraction of bound phenolics from the food matrix. Therefore, a certain extraction bias toward alkali-stable phenolic compounds cannot be excluded.

For the chokeberry pomace samples, the extraction was performed directly from dry material using the same ethanol/water solvent system (80:20, *v*/*v*) at a 1:20 (*w*/*v*) ratio, shaken for 1 h at room temperature, followed by centrifugation (10 min, 5000× *g*). The supernatant was collected and used for phenolic analysis. All extruded snacks enriched with chokeberry pomace and the chokeberry pomace sample were analyzed in triplicate.

#### 2.7.2. Total Phenolic Content

Total phenolic content (*TPC*) of the extracts obtained from the extruded snacks with incorporated chokeberry pomace and the extracted chokeberry pomace itself was determined by performing the Folin–Ciocalteu spectrophotometric assay, with modifications of previously reported protocols [26]. An aliquot of 0.10 mL of each extract was mixed with 7.90 mL of ultrapure water, followed by the addition of 0.50 mL Folin–Ciocalteu reagent. After 3 min, 1.50 mL of sodium carbonate solution (20 g/100 mL) was added to initiate the reaction. The mixtures were vortexed and kept in the dark at room temperature for 120 min, with occasional shaking. The absorbance was recorded at 750 nm using a UV–Vis spectrophotometer (Jenway 6405, Blackburn, UK). A gallic acid calibration curve was used for quantification, and the TPC results were expressed as mg gallic acid equivalents (GAE) per 100 g dry weight of the sample. Both the extruded snacks enriched with chokeberry pomace and the chokeberry pomace sample were analyzed in triplicate.

### 2.8. In Vitro Antioxidant Activity

#### 2.8.1. DPPH Radical Scavenging Assay

The DPPH radical scavenging activity was determined according to Šeregelj et al. [27], adapted to a 96-well microplate format. A volume of 250 μL of DPPH solution in methanol (0.89 mM) was mixed with 10 μL of extract and incubated in the dark at room temperature for 50 min. Absorbance was recorded at 515 nm, with methanol used as the blank. The DPPH-radical-scavenging activity was determined in triplicate (*n* = 3).

#### 2.8.2. Reducing Power

Reducing power (*RP*) was assessed using the method of Oyaizu [28], adapted for a 96-well microplate format. The sample extract (25 μL), sodium phosphate buffer (pH 6.6; 25 μL), and 1% potassium ferricyanide (25 μL) were mixed and incubated at 50 °C for 20 min. After cooling, 25 μL of 10% trichloroacetic acid was added, followed by centrifugation (2470× *g*, 10 min). The supernatant (50 μL) was mixed with distilled water (50 μL) and 0.1% ferric chloride (10 μL). Absorbance was measured at 700 nm. All measurements of *RP* were performed in triplicate and expressed as the mean ± standard deviation.

#### 2.8.3. ABTS Radical Scavenging Assay

The ABTS-scavenging activity was evaluated following the method described by Aborus et al. [29]. The activated ABTS solution (generated using MnO_2_) was mixed with 2 μL of extract and incubated at 25 °C for 35 min. Absorbance was measured at 414 nm, using distilled water as the blank. All measurements were performed in triplicate.

For all applied antioxidant assays (*DPPH*, *ABTS*, and *RP*), antioxidant activity was quantified using Trolox calibration curves, and the results were expressed as mg Trolox equivalents (TE) per 100 g dry weight (DW) of the sample. Folin–Ciocalteu reagent, 2,2-diphenyl-1-picrylhydrazyl (DPPH), 2,2′-azino-bis(3-ethylbenzothiazoline-6-sulfonic acid) (ABTS), gallic acid, Trolox, and all solvents used in the analyses were of analytical grade and purchased from Sigma-Aldrich (St. Louis, MO, USA).

### 2.9. Consumer Test of Selected Snack Samples

A hedonic study of selected snack samples (samples 0, 3, 4, 5, and 13) was performed with a panel of 104 subjects (30 men and 74 women, aged 18 to 64 years). Only fully expanded snack samples were considered appropriate for sensory analysis. Participants, including several employees from the snacks and related products industry, were selected based on the following criteria: regular consumption of any type of snacks (at least once per week), willingness to participate in the study, and absence from any food allergies. The experiment was performed in controlled laboratory conditions within isolated panel booths constructed in accordance with ISO 8589 [30]. Samples were delivered individually in the plastic cups with lids (10 g), coded with three randomly chosen numbers in a monadic way. Water was used for palate cleansing. Before the evaluation, participants received detailed instructions about the study procedure. They were informed that study consisted of two consecutive parts, a liking study and open-ended questionnaire, and that the same panel of participants would complete both parts. Participants were also informed that they could withdraw from the testing at any time if they experienced any discomfort. The study was approved by the Ethics Committee of Institute of Food Technology in Novi Sad, University of Novi Sad, Serbia (Ref. No. 175/I/8-3).

#### Liking Study

Participants were asked to evaluate samples for overall liking and liking of appearance, liking of taste, and liking of textural properties, hardness, crispiness, and adhesiveness, using a 9-point hedonic scale (1 = extremely dislike, 5 = neither like nor dislike, 9 = extremely like). In addition, participants were asked to evaluate whether the level of bitterness in the samples was appropriate by using a 9-point just-about-right (JAR) scale (1 = much too little, 3 = to little, 5 = JAR, 7 = too much, 9 = much too much).

After finishing the liking study, participants were invited to provide open-ended comments on the specific properties of each snack sample, indicating what they liked and disliked and how would they improve them. All verbal responses were subjected to qualitative analysis. To account for individual differences in wording and interpretation, frequently repeated comments were identified and semantically similar terms were grouped into broader conceptual categories. The categorization process was carried out independently by three researchers involved in the study. Categories were retained for further analysis only if they were mentioned by more than 5% of participants [31]. After independently reviewing the dataset, the researchers compared their classifications and reached a consensus on 11 final categories—most of them describing sensory attributes (taste: sweetness, saltiness and bitterness correction; flavor changes: chocolate, nutty, fruity, warm earthy spices, and general flavor comments; texture, and visual corrections), along with categories reflecting sensory defects (negative comments) and positive comments.

### 2.10. Statistical Analysis

The normality of collected data was verified using the Shapiro–Wilk test, which confirmed that the residuals followed a normal distribution, thereby fulfilling the assumptions necessary for performing Analysis of Variance (ANOVA). To quantitatively evaluate the effects of formulation factors—specifically the proportions of cinnamon and chokeberry pomace—on the extrusion-related responses (*SME*, *Torque*, *ER*, *BD*, *Hard*, *NoFract*, *Crisp work*, *Crisp index*, L*, a*, b*, C*, *WAI*, *WSI*, *FPC*, *BPC*, *TMA*, *Free DPPH*, *Bound DPPH*, *Free RP*, *Bond RP*, *Free ABTS*, and *Bound ABTS*), response surface methodology (RSM) was applied using a second-order polynomial model including linear, quadratic, and interaction terms to evaluate the effects of formulation variables (sorghum, cinnamon, and chokeberry proportions) on the responses. Model significance and adequacy were evaluated within the RSM framework. All computations were conducted using the experimental dataset in TIBCO Statistica^®^ 14.0.0.15 [32]. An artificial neural network (ANN) model was developed to complement the RSM analysis, providing a nonlinear modeling framework capable of capturing complex interactions and improving the predictive accuracy for all response variables. The ANN based on a multilayer perceptron (MLP) architecture was developed to model the relationships between processing variables and product quality responses. The input variables of the model were chokeberry pomace content, cinnamon content, extrusion temperature, and die pressure, while the output variables included the specific mechanical energy, torque, expansion ratio, bulk density, hardness, number of fractures, crisp work, crisp index, color parameters, water absorption index, water solubility index, functional properties, and antioxidant activities. The dataset was used to train, test, and validate the ANN model to ensure generalization capability. The model was trained using the Broyden–Fletcher–Goldfarb–Shanno (BFGS) algorithm with a sum-of-squares (SOS) error function. Logistic activation functions were applied in both hidden and output layers. Model performance was evaluated using standard statistical indicators to assess predictive accuracy and robustness. Qualitative data collected within the liking study were transformed into a frequency contingency table, which subsequently served as the basis for performing Correspondence Analysis (CA) to explore the associations between selected categories and the evaluated snack samples. CA was performed by using the XLSTAT software package version 2023.3.1 [33].

## 3. Results and Discussion

The *ER* is one of the most critical quality indicators for assessing extruded products. The expansion ratio (*ER*) of sorghum snack samples ranged between 3.23 and 4.58 (Table 2), with higher values observed in samples 7, 8, and 9. These samples were processed under conditions of lower feed moisture and higher die temperatures (Table 1 and Table 2), which are known to promote starch melting, rapid water vaporization, and bubble growth at the die exit. Under such conditions, the melt is able to expand more effectively before structural solidification, resulting in increased product expansion [34]. In contrast, snack samples 7 and 8 exhibited the lowest *BD* due to the highly porous internal structure of the extrudates, which associated with increased product volume despite the higher content of chokeberry pomace (Table 2). Similar trends were reported by Boakye et al. [35].

It should be noted that the experimental design was primarily structured to evaluate the effect of increasing the chokeberry pomace content, while the inclusion of cinnamon was limited to a smaller subset of samples. Consequently, the applied RSM model should be interpreted as an empirical approximation of the observed trends within the studied formulation range. Although the model provided a satisfactory fit for most responses, the estimation of interaction effects, particularly those involving cinnamon, may be less reliable due to the structure of the experimental design.

The color attributes (L*, a*, b*, and C*) were primarily influenced by the chokeberry pomace content in the formulation (Table 2).

It should be noted that variations in feed moisture (water addition) among formulations may have influenced extrusion responses, and therefore, effects cannot be attributed solely to the formulation composition. The *WAI* and *WSI* are useful parameters for predicting the post-processing behavior of extruded products, i.e., during rehydration, storage, and consumption. The *WAI* describes the water-binding ability of starch and fiber, whereas *WSI* indicates the release of soluble components from macronutrients [36,37]. In the present study, *WAI* decreased from 6.89 (g/g) in sample 0 to values around 4.2 (g/g)–5.1 (g/g) in samples 2–13 (Table 2), indicating reduced water-binding capacity, whereas *WSI* increased from 26.83 (g/100 g) to above 30 (g/100 g) in most samples, suggesting enhanced solubilization of macronutrient components during extrusion.

**Figure 1:**
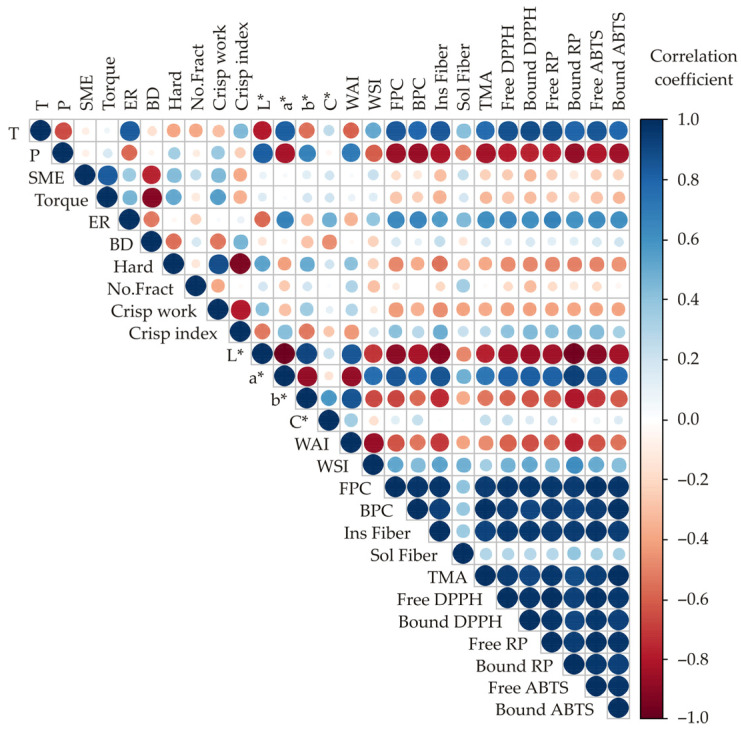
Colored correlation matrix among the processing variables, physical attributes, color parameters, and antioxidant indicators. The size of the circle represents the absolute value of the correlation coefficient.

The maximum force registered during fracture of the tested samples (hardness of the sorghum-based extruded snacks) ranged from 51.62 N to 71.75 N (Table 2). Crispiness was also influenced: the *NoFract* during the compression ranged from 62.73 to 119.15, the *Crisp index* between 0.32 × 10^−3^ and 0.58 × 10^−3^, and *Crisp work* between 2.95 and 5.29 Nmm. The analysis of the correlation matrix (Figure 1) showed that temperature and pressure were less correlated to the sorghum-based extruded snacks texture. On the other hand, higher torque was associated with the development of harder and less crispy structures (crispiness work *r* = 0.55, *p* < 0.05). Moreover, the insoluble fibers exerted similar influence on texture development. Higher levels of insoluble fiber tended to reduce the hardness of sorghum-based extruded snacks (*r* = 0.549, *p* < 0.05). Soluble fibers were also associated with softer, crispier structures, but the correlations were less strong and not significant.

These findings partially agree with previous reports indicating that the mechanical behavior of extruded products is governed by characteristics of both the continuous phase (viscosity, cell wall thickness) and the dispersed phase (e.g., porosity, cell size, density, and their distribution) and is largely driven by starch–fiber interactions [38]. Insoluble dietary fibers are generally reported to reduce sectional expansion, increase density, and promote the formation of numerous small pores with thick walls, resulting in harder, more rigid extrudates [38,39]. However, our results support observations from other studies reporting opposite trends, where the incorporation of insoluble fibers reduced hardness [39]. Reduced hardness may arise from weakened interactions or adhesion between polysaccharides and proteins in the presence of insoluble fibers [39,40,41]. The inconsistent outcomes regarding the influence of fibers on the extrudate texture could be due to different water-absorption properties and viscoelastic properties of fibers.

Soluble fibers such as inulin and guar gum have been shown to improve extrudate texture by providing a higher expansion volume [38,42]. Extrudates from corn starch and with high methoxyl pectin (soluble fiber) were improved in quality and showed higher expansion [43]. Given that chokeberry pomace contains insoluble (cellulose, hemicellulose, lignin) and soluble fibers (pectin) [44], its incorporation likely contributed to the more favorable textural properties observed.

Table 2 shows that increasing the proportion of chokeberry pomace was accompanied by a significant linear increase in both the free phenolic content (*FPC*) and bound phenolic content (*BPC*), accompanied by a simultaneous increase in insoluble and soluble dietary fiber fractions. The increase in *BPC* with higher pomace levels may indicate strong interactions between phenolic compounds and the fiber matrix, which are typical for fruit-pomace-enriched cereal extrudates [14,45,46]. Similar observations were reported for chokeberry-based products, where fiber-associated phenolics contributed substantially to the antioxidant potential of the final products [13]. The increased phenolic content was reflected in higher antioxidant activity values determined via DPPH, *RP*, and ABTS assays (Table 2). Anthocyanins are known to be heat-sensitive compounds, and extrusion processing may promote their degradation through thermal, oxidative, and mechanical effects. Nevertheless, detectable anthocyanin levels were retained after processing, indicating partial preservation of these compounds despite extrusion conditions. Free antioxidant fractions showed a more pronounced increase compared to bound fractions, suggesting the enhanced release and extractability of phenolic compounds during extrusion, while a considerable proportion remained associated with the fiber matrix. Strong correlations among antioxidant assays were observed, which is consistent with the related redox responses measured by these in vitro methods and the increased phenolic content of the investigated samples, as previously reported for cereal-based pomace extrudates [47,48]. Cinnamon addition further enhanced antioxidant activity, particularly in formulations containing moderate chokeberry levels (8–13%), suggesting a possible synergistic interaction between phytochemicals originating from both plant materials [49]. Overall, the results indicate that chokeberry pomace enrichment, combined with balanced cinnamon addition, improved the phenolic content and antioxidant potential of sorghum extruded snacks.

### 3.1. Correlation Analysis

The correlation analysis (Figure 1) revealed several significant relationships among processing conditions, physicochemical properties, texture, color, and bioactive compounds (*p* < 0.05). Temperature was positively associated with the expansion ratio (*ER*), phenolic compounds, insoluble fiber, and antioxidant activities, while negatively correlated with pressure (*P*), lightness (L*), yellowness (b*), and *WAI*. In contrast, *P* showed opposite trends, being negatively associated with *ER* and bioactive-related parameters, but positively correlated with L*, b*, and *WAI*. These findings indicate that higher extrusion temperatures promoted the expansion and retention of bioactive compounds, whereas increased pressure contributed to lighter products with lower functional quality [37,50,51,52,53,54,55,56].

*SME* and *Torque* were positively correlated, while both parameters were negatively associated with bulk density (*BD*), confirming that greater mechanical energy input promoted expansion and reduced product compactness. *ER* was negatively correlated with hardness (*Hard*) and positively correlated with crispness, *WAI*, *WSI*, phenolic compounds, and antioxidant activities, indicating that expanded products exhibited improved texture and functional properties [37,51,52,53,54,55,56].

Strong interrelationships were also observed among color and bioactive parameters. L* was negatively correlated, while a* was positively correlated, with phenolic compounds, insoluble fiber, and antioxidant activities, suggesting that darker and redder samples contained higher levels of bioactive compounds. Furthermore, phenolic compounds, dietary fiber, and antioxidant activity assays were all strongly positively correlated with each other, highlighting the close association between phenolic content and antioxidant potential in the extruded samples [37,50,51,52,53,54,55,56,57].

The correlation matrix clearly indicates that both temperature and pressure are dominant factors influencing not only the physical attributes (*ER*, *BD*, *Hard*) but also the color development and antioxidant potential of the products. Elevated temperatures favored the formation of darker, more intensely colored products with enhanced free and bound antioxidant activities, likely due to increased phenolic release, pigment formation, and Maillard-reaction-derived antioxidants [58]. Conversely, higher pressures promoted denser structures with reduced pigment intensity and lower antioxidant activity [45]. These findings collectively demonstrate the critical balance among process conditions, structural modifications, and functional properties in determining the overall quality and antioxidant potential of the extruded samples.

### 3.2. Response Surface Methodology (RSM) Analysis

A second-order polynomial model was applied to evaluate the individual and combined effects of chokeberry pomace and cinnamon powder levels on the technological, physical, and functional properties of the extruded products. The adequacy of each model was assessed based on the coefficient of determination (*R*^2^) and the adjusted coefficient (adj. *R*^2^), while the significance of linear, quadratic, and interaction terms was evaluated using the corresponding *p*-values. The model included the linear and quadratic effects of chokeberry pomace content, cinnamon addition, and their interaction term (chokeberry × cinnamon). The significance of individual model terms was evaluated using Analysis of Variance (ANOVA), and statistically significant effects were identified at *p* < 0.05. This modeling approach enabled the assessment of the main and interaction effects of formulation variables on product quality attributes (Table 3).

The effects of chokeberry and cinnamon contents on color, texture, water-binding, and antioxidant-related responses were analyzed using response surface methodology (RSM). The linear effect of chokeberry content was significant for the following: *FPC*, *BPC*, *Ins Fibre*, *Sol Fibre*, *Free DPPH*, *Bound DPPH*, *Free RP*, *Bound RP*, *Free ABTS*, and *Bound ABTS*. This indicates that increasing the chokeberry content has a direct, proportional effect on the functional properties, fiber content, and antioxidant-related responses. The quadratic effect of chokeberry pomace content was significant for the following: L*, a*, b*, *WAI*, *WSI*, *FPC*, *Sol Fibre*, *Bound DPPH*, and *Bound RP*. This shows non-linear effects for these responses, suggesting that moderate chokeberry pomace content levels optimize these properties, while very low or high content may reduce them.

The linear effect of cinnamon content was significant for the following: *FPC*, *BPC*, *Sol Fiber*, and Free *RP*. This indicates a linear influence of cinnamon on specific antioxidant/functional properties. The quadratic effect of cinnamon was not significant for any response.

Significant interaction terms (Chokeberry × Cinnamon) were observed for the following: *FPC*, *BPC*, and *Sol Fiber*. This suggests that the combination of chokeberry pomace and cinnamon powder affects the specific antioxidant/functional properties, indicating synergistic interactions.

High *R*^2^ values were observed for antioxidant-related responses (Free/Bound DPPH, Free/Bound RP, Free/Bound ABTS), indicating excellent model fit.

#### Verification of RSM Models

Verification of the RSM models demonstrated satisfactory agreement between predicted and experimental values for most responses, confirming the adequacy of the developed models (Table 4).

The coefficient of determination (*R*^2^) values ranged from 0.289 to 0.998, while RMSE values varied between 0.006 (*BD*) and 18.811 (*TMA*), indicating generally good agreement between predicted and observed data. Mean bias error (MBE) values were close to zero for all responses, confirming the absence of systematic prediction bias. Mean percentage error (MPE) ranged from 1.408% (*T*) to 288.647% (*TMA*), while AARD values remained below 6% for most responses.

High predictive accuracy was observed for *FPC*, *BPC*, *Ins Fiber*, and antioxidant parameters, including *Bound DPPH*, *Free RP*, *Bound RP*, *Free ABTS*, and *bound ABTS*. Acceptable fits were obtained for the color parameters L*, a*, b*, *WAI*, and *WSI*. Lower predictive performance was observed for *BD*, *Crisp work*, *Torque*, and *TMA*, indicating higher variability in these responses. The RSM models demonstrated satisfactory predictive capability with low error metrics for most responses, confirming their suitability for process and quality prediction.

### 3.3. Artificial Neural Network Model

The artificial neural network (ANN) model based on a multilayer perceptron (MLP 4-4-25) architecture exhibited excellent predictive performance across most responses. The model was trained using the BFGS 41 algorithm with a sum of squares (SOS) error function, employing logistic activation functions in both hidden and output layers. The overall training, testing, and validation performances were 0.778, 0.166, and 0.857, respectively, with corresponding errors of 657.770, 318.794, and 343.958, indicating efficient convergence and consistent generalization of the network.

The coefficient of determination (*R*^2^) confirmed strong model performance, with very good prediction accuracy achieved during testing and validation for all responses, indicating excellent agreement between predicted and experimental values. During training, high *R*^2^ values were obtained for most responses, including *FPC*, *BPC*, *Ins Fiber*, *WAI*, *Bound DPPH*, *Free RP* (0.990), *Bound RP* (0.969), *Free ABTS* (0.968), and *Bound ABTS* (0.974), demonstrating strong learning of nonlinear relationships.

Moderate to high performance was observed for color parameters (L*, a*, b*, C*) and functional indices (*ER*, *WSI*), with *R*^2^ values ranging from 0.879 to 0.966. Lower accuracy was obtained for *Crisp work* (*R*^2^ = 0.357), *NoFract* (*R*^2^ = 0.489), and *Hard* (*R*^2^ = 0.650), indicating higher variability. The poorest fit was observed for *TMA* (*R*^2^ = 0.007), suggesting limited model capability for this response, likely due to high noise or complex nonlinear behavior.

The ANN model demonstrated outstanding predictive ability across most physicochemical, functional, and antioxidant properties, with minimal error and high *R*^2^ values in testing and validation stages. These results confirm the suitability of the MLP 4-4-25 network for accurate modeling of complex response patterns, significantly outperforming traditional RSM models in predictive precision and generalization [59].

It should be noted that the ANN modeling in this study was performed using a relatively small dataset, which inherently limits the generalization capacity and robustness of the network [24]. The restricted number of experimental samples can lead to overfitting, where the model learns specific data patterns rather than the underlying functional relationships, resulting in artificially high *R*^2^ values during testing and validation [60]. Therefore, this study does not aim to establish a fully optimized or deployable machine learning model, but rather to demonstrate the potential of ANN as a tool for identifying and visualizing nonlinear trends within the extrusion process. The applied network serves as an exploratory approach to capture response tendencies and process interactions rather than to construct a predictive system for industrial application.

#### Verification of ANN Models

The verification of the ANN model confirmed a high degree of accuracy and predictive reliability for most responses (Table 5). The coefficient of determination (*R*^2^) values ranged from 0.109 to 0.996, with the majority exceeding 0.90, indicating a strong correlation between predicted and experimental values. The root mean square error (RMSE) varied between 0.004 (*BD*) and 28.584 (*TMA*), demonstrating low deviation across most responses. Mean bias error (MBE) values were close to zero for nearly all outputs (ranging from –2.333 to 1.394), confirming the absence of systematic over- or underestimation. Similarly, mean percentage error (MPE) and average absolute relative deviation (AARD) values were generally below 10%, suggesting acceptable prediction accuracy for the majority of responses. The sum of squared errors (*SSE*) ranged from 0.000 (*BD*) to 11,438.479 (TMA), indicating low residual variation for most predicted properties.

Excellent agreement between predicted and measured values was obtained for physicochemical and functional parameters, including *FPC*, *BPC*, *Ins Fiber*, and *WAI*, with low prediction errors across all metrics. Comparable performance was observed for antioxidant responses (*Free* and *Bound DPPH*, *RP*, and *ABTS*), with R^2^ values ranging from 0.965 to 0.983.

Color parameters (L*, a*, b*, C*) also showed strong predictive ability (*R*^2^ = 0.912–0.978), while structural parameters such as the expansion ratio and torque were modeled with satisfactory accuracy. Lower performance was observed for texture-related variables, including *Hard*, *NoFract*, *Crisp work*, and *Crisp index*, reflecting higher variability in these responses. The poorest prediction was obtained for *TMA*, indicating limited model capability for this parameter. The ANN verification results confirmed excellent predictive accuracy and low error for the majority of physicochemical, functional, and antioxidant parameters, highlighting the reliability and robustness of the developed MLP model in simulating nonlinear relationships between formulation and response variables.

A comparative analysis between the ANN and RSM models revealed notable differences in predictive performance depending on the response variable. In general, both models achieved high coefficients of determination (*R*^2^), indicating satisfactory fit to the experimental data; however, the ANN model demonstrated superior predictive ability for several complex, nonlinear responses. Significant improvements were observed for *Torque* (Δ*R*^2^ = +0.315), *ER* (Δ*R*^2^ = +0.263), *BD* (Δ*R*^2^ = +0.461), and color saturation (C*, Δ*R*^2^ = +0.403), suggesting that the ANN model more effectively captured the nonlinear and interactive relationships inherent in the extrusion process. Moderate advantages were also recorded for b* (Δ*R*^2^ = +0.061), *WAI* (Δ*R*^2^ = +0.082), and *Crisp work* (Δ*R*^2^ = +0.052), further confirming the ANN’s enhanced capacity for modeling complex response behavior.

Conversely, the RSM model exhibited slightly better performance for a subset of responses, including *WSI* (Δ*R*^2^ = –0.101), *Sol Fiber* (Δ*R*^2^ = –0.146), and *TMA* (Δ*R*^2^ = –0.539), as well as some antioxidant parameters (*Free RP*, *Bound DPPH*, and *Free ABTS*), though the magnitude of these differences was generally small (|Δ*R*^2^| < 0.03). Both models produced nearly identical results for the color parameters L* and a* and for compositional attributes such as *FPC* and *BPC*, indicating stable and consistent predictions independent of the modeling approach.

RSM adequately described the linear and quadratic relationships between process variables and responses, and the ANN model provided a more flexible and accurate representation of nonlinear dependencies, especially for mechanical and physical properties that are highly sensitive to subtle changes in extrusion conditions. This comparison highlights the ANN’s greater adaptability in modeling complex systems, though its performance is contingent on data availability and network training stability.

Despite the valuable insights obtained, several methodological limitations should be acknowledged. The study was based on a relatively small number of formulations (*n* = 14), which restricts the statistical power of modeling approaches and limits the generalization of the obtained results. In addition, the experimental design was not fully balanced with respect to processing parameters, as variations in feed moisture (water addition) occurred among selected formulations. Since moisture is a critical factor influencing expansion behavior, texture development, and nutrient retention in the extrusion processes, its simultaneous variation may have introduced confounding effects that prevent the exclusive attribution of observed changes to the chokeberry pomace concentration. Furthermore, the applied RSM and ANN models should therefore be interpreted as data-driven, exploratory tools rather than fully predictive or mechanistic models. These limitations highlight the need for more controlled experimental designs with larger datasets in future studies to enable more robust statistical inference and model generalization.

### 3.4. Consumer Study

**Figure 2:**
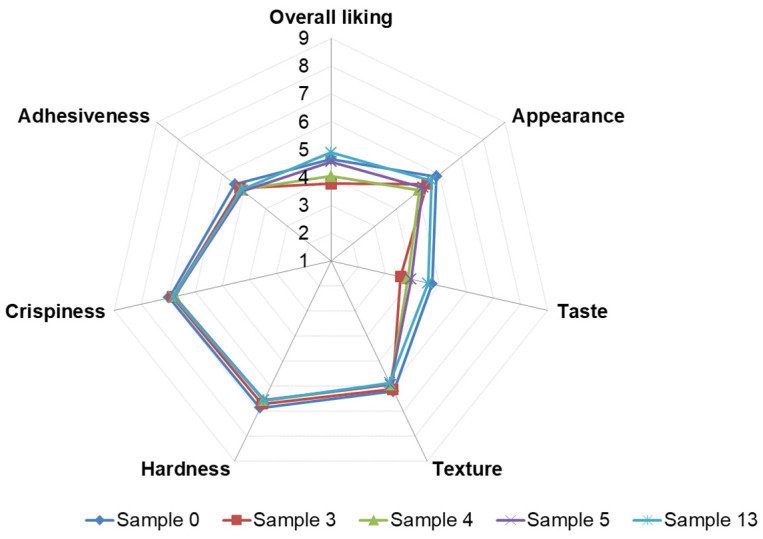
Results of the liking study for snack samples.

The consumer study was conducted to better understand consumers’ liking patterns and to identify key sensory drivers that could guide further optimization of product properties [61]. The obtained results revealed that none of the analyzed samples achieved clear consumer acceptance, as overall liking scores for all samples remained below the neutral value of 5 (Figure 2). The highest overall liking was recorded for the sample containing 13% chokeberry pomace with cinnamon (4.89), whereas the lowest score was observed for Sample 3 (3.77). The control sample (Sample 0) and Samples 4 and 5 showed similar levels of overall liking (4.05–4.65), suggesting generally limited acceptability across formulations. An analysis of liking scores for individual sensory modalities (appearance, taste, and texture) indicated that taste was the most critical attribute limiting the overall acceptance of the samples. Andersen et al. [62] showed that liking of taste (understood as flavor) is the most important sensory modality for evaluating overall liking, indicating that consumers primarily pay attention to product taste. In the present study, taste scores were below 5 for all samples, with the lowest values observed for the Samples 3 and 4 (3.57 and 3.84, respectively). Although Sample 13 showed a slight improvement in taste perception (4.58), this value still did not reach the neutral liking level. In contrast, appearance and texture-related attributes (texture, hardness, and crispiness) were rated above the neutral point for all samples. In particular, crispiness and hardness received the highest scores, ranging from approximately 6.6 to 7.0, indicating that the textural properties of the products were well perceived and represented a clear strength of the formulations.

**Figure 3:**
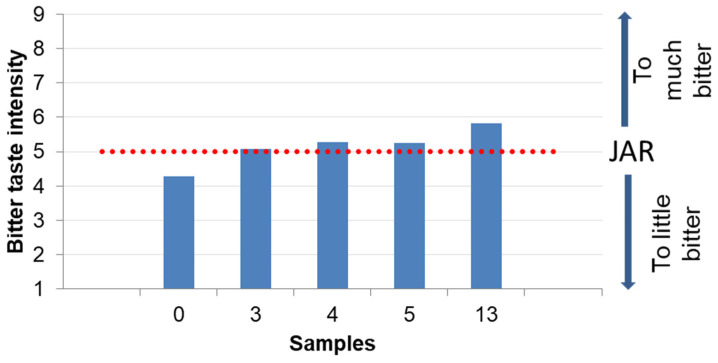
Consumer perception of bitter taste intensity in relation to the optimal JAR level.

The application of the just-about-right (JAR) scale for the evaluation of bitter taste intensity revealed clear differences among the samples in relation to the optimal bitterness level (JAR = 5), indicated by the red dashed line (Figure 3). Sample 0 was positioned below the JAR zone, suggesting that bitterness was perceived as too weak and likely insufficient for consumers. Samples 3, 4, and 5 were very close to the optimal JAR value, indicating that their bitter taste intensity most closely matched consumer expectations. In contrast, Sample 13 was located above the JAR line, indicating a slightly more intense bitter taste, which may negatively affect product acceptance. Overall, these results suggest that the optimal bitterness level was achieved in Samples 3, 4 and 5, whereas formulations of Samples 0 and 13 would require adjustment to improve sensory acceptability.

#### Correspondence Analysis

**Figure 4:**
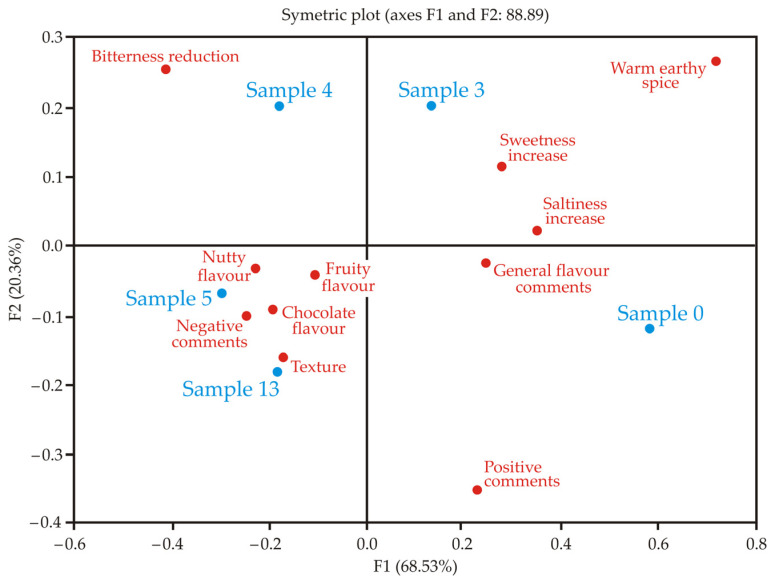
Correspondence analysis of snack samples and comment categories cited by consumers.

Correspondence analysis (CA) was applied to examine the relationships between snack samples and the qualitative comment categories. It provides valuable insight into the drivers underlying the hedonic responses observed in the liking study. The results are presented in Figure 4. In total, 415 comments were collected during the qualitative analysis and subsequently grouped into 11 categories referring to sensory attributes related to taste (sweetness, saltiness and bitterness corrections), flavor (chocolate, nutty, fruity, warm earthy spices and general flavor comments), texture, and visual corrections, along with categories reflecting sensory defects (negative comments) and positive comments. The distribution of comments differed significantly among samples (*χ*^2^ = 56.085, *p* < 0.05), indicating that the information provided had a significant impact on consumers’ perception of the snack samples. The variables were projected onto a two-dimensional factor space, with the first two dimensions explaining approximately 89% of the total variance, indicating a very good representation of the data structure. The first dimension (F1) clearly separates samples based on flavor comments. Sample 0 is placed on the positive side of this dimension and associated with positive comments and general flavor comments, suggesting a relatively neutral and broadly acceptable flavor profile. This positioning indicates that the control formulation did not elicit strong sensory expectations but also highlights potential for targeted flavor enhancement.

In contrast, samples located on the negative side of F1 (Samples 5 and 13) were associated with negative comments and with specific flavor descriptors such as chocolate flavor, nutty flavor, fruity flavor, and warm earthy spice. In the context of the qualitative analysis, these descriptors should not be interpreted as existing flavor notes, but rather as consumer-driven suggestions for product improvement, indicating that the addition of such flavors or spices could enhance flavor complexity and acceptance. At the same time, texture-related comments suggest that textural properties require further adjustment.

The second dimension (F2), explaining 20.36% of the variance, reflects differences related to taste modulation and flavor balance. Sample 4 was associated with bitterness reduction and indicates that consumers perceived bitterness as a limiting factor and that reducing bitter notes represents a key direction for improving acceptance. Sample 3 was positioned near sweetness increases and saltiness increases; this suggests that consumers identified the need for enhancing sweetness and/or saltiness to achieve a more balanced flavor profile for this formulation.

## 4. Conclusions

The enrichment of sorghum-based extruded snacks with chokeberry pomace significantly improved their nutritional and functional properties, particularly by increasing total monomeric anthocyanins (up to 25.12 mg/100 g at 30% pomace) and antioxidant activity, with the highest bioactivity observed in formulations containing 27–30% pomace. The addition of 1% cinnamon further enhanced antioxidant activity compared to corresponding formulations without cinnamon.

Chokeberry pomace influenced key technological properties, contributing to a crispier more brittle texture due to the increased dietary fiber content, especially insoluble fractions. Both RSM and ANN models effectively predicted the influence of formulation variables on the above key parameters, demonstrating the value of advanced modeling approaches for snack product development.

Hedonic analyses indicate that overall consumer acceptance of the analyzed snack samples was primarily limited by insufficient taste and flavor intensity, while textural properties such as crispiness and hardness were generally well accepted.

Based on the combined physicochemical and sensory results, an optimal formulation range of approximately 10–13% chokeberry pomace can be recommended, provided that further improvements in flavor are achieved through bitterness reduction and the optimization of sweetness and saltiness, thereby balancing enhanced bioactivity with acceptable structural and sensory properties.

From an industrial perspective, these findings highlight the potential of chokeberry pomace as a sustainable functional ingredient for the development of fiber-rich and antioxidant-enriched extruded snacks, supporting the valorization of juice-processing by-products in functional food production.

## Figures and Tables

**Table 1:** Dry mixes composition and the extrusion process parameter inputs and outputs.

Label of Trial	0	1	2	3	4	5	6	7	8	9	10	11	12	13
White sorghum (%)	100	98	95	92	90	87	84	80	77	73	70	91	89	86
Chokeberry pomace (%)	0	2	5	8	10	13	16	20	23	27	30	8	10	13
Cinnamon (%)	0	0	0	0	0	0	0	0	0	0	0	1	1	1
Dry mixes														
Moisture content ^a^ (%)	10.02	10.12	10.25	10.39	10.48	10.62	10.75	10.94	11.07	11.26	11.39	10.39	10.48	10.62
Extrusion														
Temperature ^b^ (°C)														
Section 3 of the extruder barrel	75.50	75.50	75.50	75.50	75.50	75.50	75.50	75.50	75.40	75.40	75.50	75.80	75.60	75.80
Section 6 of the extruder barrel	111.2	113.4	113.7	114.2	114.1	114.9	114.7	114.7	115.3	115.1	114.2	114.4	114.5	114.4
Die	159.0	161.0	161.0	163.0	167.0	169.0	170.0	177.0	184.0	185.0	174.0	172.0	172.0	174.0
Screw speed (RPM)	900	900	900	900	900	900	900	900	900	900	900	900	900	900
Die diameter (mm)	4	4	4	4	4	4	4	4	4	4	4	4	4	4
Open die area (mm^2^)	12.56	12.56	12.56	12.56	12.56	12.56	12.56	12.56	12.56	12.56	12.56	12.56	12.56	12.56
Throughput (kg/h)	50	50	50	50	50	50	50	50	50	50	50	50	50	50
Water throughput (kg/h)	2.40	2.20	2.20	2.20	2.20	2.20	2.20	0.00	0.00	0.00	2.00	2.20	2.20	2.20
Moisture content ^c^ (%)	14.14	13.91	14.03	14.17	14.25	14.39	14.51	10.94	11.07	11.26	14.08	14.17	14.25	14.39
Die pressure (MPa)	0.08	0.07	0.05	0.02	0.03	0.03	0.04	0.03	0.01	0.00	0.00	0.04	0.06	0.06
Torque ^d^ (Nm)	114.4	118.8	118.8	118.8	118.8	114.4	112.2	121.0	123.2	118.8	99.0	114.4	118.8	114.4
SME ^e^ (Wh/kg)	145.3	148.1	150.1	163.0	157.8	145.3	143.4	154.7	156.6	149.1	124.6	137.6	142.9	137.3

^a^ Moisture content determined after conditioning by rapid moisture analyzer; ^b^ measured by sensors located in the extruder barrel; ^c^ total moisture content during extrusion calculated based on moisture content of dry mixes, material throughput, and water throughput in the extruder barrel; ^d^ motor load, 100% torque is 220 Nm; ^e^ specific mechanical energy.

**Table 2:** Experimental results.

**Label of Trial**	**Raw Material Composition (%)**			**Process Output**				**Product Output**		
	**Sorghum**	**Cinnamon**	**Chokeberry**	***T***	***P***	***SME***	***Torque***	***ER***	***BD***	***Hard***
**0**	100	0	0	159	0.08	145.3	114.4	3.23 ± 0.1497	0.05 ± 0.0002	66.66 ± 7.27
**1**	98	0	2	161	0.07	148.1	118.8	3.29 ± 0.1474	0.05 ± 0.0010	68.55 ± 6.57
**2**	95	0	5	161	0.05	150.1	118.8	3.29 ± 0.2091	0.05 ± 0.0003	71.75 ± 7.61
**3**	92	0	8	163	0.02	163.0	118.8	3.53 ± 0.2492	0.05 ± 0.0007	64.04 ± 5.91
**4**	90	0	10	167	0.03	157.8	118.8	3.36 ± 0.1503	0.05 ± 0.0002	59.76 ± 6.91
**5**	87	0	13	169	0.03	145.3	114.4	3.60 ± 0.2024	0.06 ± 0.0007	58.91 ± 5.18
**6**	84	0	16	170	0.04	143.4	112.2	3.57 ± 0.2577	0.06 ± 0.0004	57.39 ± 5.61
**7**	80	0	20	177	0.03	154.7	121.0	4.06 ± 0.2533	0.04 ± 0.0006	64.34 ± 6.49
**8**	77	0	23	184	0.01	156.6	123.2	4.50 ± 0.2711	0.04 ± 0.0001	59.84 ± 5.79
**9**	73	0	27	185	0.00	149.1	118.8	4.58 ± 0.2054	0.05 ± 0.0004	64.83 ± 5.40
**10**	70	0	30	174	0.00	124.6	99.0	3.26 ± 0.1935	0.07 ± 0.0007	55.04 ± 2.91
**11**	91	1	8	172	0.04	137.6	114.4	3.35 ± 0.1113	0.05 ± 0.0003	59.55 ± 5.10
**12**	89	1	10	172	0.06	142.9	118.8	3.52 ± 0.1814	0.05 ± 0.0015	68.62 ± 5.06
**13**	86	1	13	174	0.06	137.3	114.4	3.54 ± 0.1684	0.06 ± 0.0004	51.62 ± 6.28
Label of trial	Product output									
	*NoFract*	*Crisp work*	*Crisp index*	L*	a*	b*	C*	*WAI*	*WSI*	*FPC*
0	112.20 ± 10.45	4.22 ± 0.56	0.37 ± 0.0700	74.31 ± 0.8787	5.20 ± 0.1306	16.34 ± 0.3615	17.14 ± 0.3748	6.89 ± 0.3362	26.83 ± 0.3556	32.58 ± 0.9600
1	106.20 ± 8.24	4.54 ± 0.43	0.33 ± 0.0600	58.89 ± 1.22	8.17 ± 0.3100	11.60 ± 0.4987	14.19 ± 0.5417	6.48 ± 0.9932	28.11 ± 0.1932	40.76 ± 0.9900
2	107.60 ± 8.55	4.74 ± 0.59	0.32 ± 0.0700	49.17 ± 0.7399	9.87 ± 0.1853	9.30 ± 0.2082	13.56 ± 0.2726	4.99 ± 0.1606	28.78 ± 0.0759	51.61 ± 1.97
3	106.50 ± 10.41	4.27 ± 0.62	0.41 ± 0.0800	40.29 ± 1.62	11.60 ± 0.3310	8.77 ± 0.2203	14.55 ± 0.3822	4.29 ± 0.0555	32.34 ± 0.0820	83.46 ± 1.85
4	119.55 ± 9.19	3.49 ± 0.52	0.55 ± 0.1100	39.16 ± 1.53	10.92 ± 0.3146	7.99 ± 0.1261	13.53 ± 0.3220	4.82 ± 0.0141	30.19 ± 0.7578	94.78 ± 0.4900
5	105.00 ± 7.44	3.81 ± 0.42	0.47 ± 0.0600	35.84 ± 1.29	11.55 ± 0.2009	7.65 ± 0.1009	13.85 ± 0.2159	4.71 ± 0.3050	30.60 ± 0.2985	101.36 ± 1.59
6	112.70 ± 12.23	3.50 ± 0.54	0.58 ± 0.1400	31.26 ± 0.3233	11.61 ± 0.1896	7.08 ± 0.2144	13.60 ± 0.2606	5.07 ± 0.0743	29.56 ± 0.7929	134.10 ± 1.97
7	99.91 ± 6.55	4.09 ± 0.62	0.41 ± 0.1100	30.12 ± 2.32	13.09 ± 0.1638	8.61 ± 0.1410	15.67 ± 0.1914	4.50 ± 0.0368	32.17 ± 0.2020	150.15 ± 0.4000
8	104.89 ± 12.37	3.75 ± 0.54	0.50 ± 0.0900	27.46 ± 1.23	13.40 ± 0.2844	8.35 ± 0.2347	15.79 ± 0.3599	4.41 ± 0.1802	31.74 ± 0.1077	158.00 ± 3.95
9	90.20 ± 4.78	4.09 ± 0.33	0.46 ± 0.0900	26.76 ± 1.02	13.88 ± 0.1158	8.10 ± 0.1193	16.07 ± 0.1423	4.75 ± 0.4117	29.74 ± 0.5654	175.63 ± 2.61
10	104.60 ± 11.63	2.93 ± 0.18	0.53 ± 0.0500	24.49 ± 1.82	12.60 ± 0.8459	6.63 ± 0.4565	14.24 ± 0.9571	4.21 ± 0.0298	30.73 ± 0.0028	189.31 ± 2.76
11	100.40 ± 14.04	2.95 ± 0.32	0.54 ± 0.1200	41.43 ± 1.53	11.04 ± 0.2923	8.84 ± 0.2986	14.14 ± 0.4065	4.46 ± 0.1128	31.06 ± 0.3344	83.83 ± 1.17
12	62.73 ± 5.37	5.29 ± 0.69	0.41 ± 0.0700	40.96 ± 2.13	11.08 ± 0.5124	8.41 ± 0.3400	13.91 ± 0.6081	4.48 ± 0.1406	31.56 ± 0.0835	88.10 ± 0.0100
13	88.50 ± 5.54	3.11 ± 0.49	0.70 ± 0.1700	37.02 ± 0.5225	11.53 ± 0.3701	8.05 ± 0.2452	14.06 ± 0.4206	4.67 ± 0.0284	29.50 ± 0.5298	94.09 ± 3.54
**Label of trial**	**Product output**									
	*BPC*	*Ins Fiber*	*Sol Fiber*	*TMA*	*Free DPPH*	*Bound DPPH*	*Free RP*	*Bound RP*	*Free ABTS*	*Bound ABTS*
0	14.53 ± 0.05	7.61 ± 1.90	0.16 ± 0.0400	nd	3.45 ± 0.00	0.91 ± 0.0000	1.25 ± 0.00	0.03 ± 0.0000	3.12 ± 0.00	0.51 ± 0.0000
1	18.01 ± 0.01	8.54 ± 2.14	0.17 ± 0.0420	1.51 ± 0.00	4.06 ± 0.00	1.24 ± 0.00	1.99 ± 0.00	0.06 ± 0.0000	7.54 ± 0.64	2.13 ± 0.00
2	24.35 ± 0.36	10.35 ± 2.59	0.28 ± 0.0700	2.98 ± 0.03	4.97 ± 0.01	1.25 ± 0.00	2.23 ± 0.00	0.09 ± 0.0000	8.01 ± 0.32	2.56 ± 0.00
3	31.17 ± 0.11	11.22 ± 2.81	0.37 ± 0.0920	3.28 ± 0.00	6.56 ± 0.31	1.98 ± 0.02	2.97 ± 0.01	0.11 ± 0.0000	10.56 ± 1.14	3.75 ± 0.00
4	37.04 ± 0.00	13.63 ± 3.41	0.47 ± 0.1170	5.78 ± 0.02	7.25 ± 0.02	2.03 ± 0.00	3.06 ± 0.00	0.11 ± 0.0000	11.57 ± 0.36	4.09 ± 0.01
5	42.86 ± 1.15	14.74 ± 3.69	0.71 ± 0.1770	8.23 ± 1.41	8.47 ± 0.04	2.41 ± 0.00	3.69 ± 0.02	0.12 ± 0.0000	14.09 ± 1.98	5.69 ± 0.00
6	50.02 ± 1.62	15.40 ± 3.85	0.62 ± 0.1550	10.12 ± 0.69	10.11 ± 0.85	3.04 ± 0.01	4.71 ± 0.26	0.13 ± 0.0000	17.57 ± 0.55	7.11 ± 0.03
7	64.21 ± 0.33	16.88 ± 4.22	0.62 ± 0.1550	13.60 ± 1.32	14.83 ± 2.90	3.76 ± 0.00	6.05 ± 0.03	0.15 ± 0.0000	20.41 ± 0.09	8.18 ± 0.00
8	71.12 ± 2.47	17.18 ± 4.30	0.82 ± 0.2050	17.91 ± 0.85	15.36 ± 1.47	4.17 ± 0.00	7.13 ± 0.02	0.16 ± 0.0000	21.32 ± 0.71	10.65 ± 0.00
9	78.52 ± 1.78	19.10 ± 4.77	0.40 ± 0.1000	21.66 ± 2.31	16.98 ± 0.71	4.83 ± 0.01	7.56 ± 0.02	0.16 ± 0.0000	23.66 ± 0.30	11.06 ± 0.01
10	83.64 ± 4.23	21.13 ± 5.28	0.27 ± 0.0670	25.12 ± 0.36	18.65 ± 0.02	5.24 ± 0.25	8.13 ± 0.00	0.18 ± 0.0100	25.98 ± 1.01	13.59 ± 0.02
11	25.11 ± 0.01	13.14 ± 3.29	0.77 ± 0.1920	2.74 ± 0.00	7.65 ± 0.03	2.76 ± 0.03	2.97 ± 0.02	0.09 ± 0.0000	10.73 ± 0.01	2.91 ± 0.00
12	26.69 ± 0.12	13.61 ± 3.40	0.11 ± 0.0270	3.11 ± 0.01	8.01 ± 0.01	2.97 ± 0.12	3.41 ± 0.00	0.10 ± 0.0000	12.69 ± 0.05	3.38 ± 0.01
13	30.58 ± 0.44	14.49 ± 3.62	0.12 ± 0.0300	5.36 ± 0.00	10.79 ± 0.46	3.06 ± 0.01	4.93 ± 0.06	0.12 ± 0.0000	15.33 ± 1.36	4.75 ± 0.01

*T*—die temperature (°C); *P*—pressure at the die (MPa); *SME*—specific mechanical energy (Wh/kg); *Torque* (Nm); *ER*—expansion ratio; *BD*—bulk density (g/mL); *Hard*—extrudate hardness (kg). *NoFract*—crispness by number of fractures; *Crisp work*—Crispiness work (Nmm); *Crisp index*—Crispiness index (×10^−3^); L*—brightness from black to white; a*—(+/−) value indicates red/green chromaticity; b*—(+/−) value denotes yellow/blue chromaticity; C*—value assesses color intensity and saturation; *WAI*—water absorption index (g/g); *WSI*—water solubility index (g/100 g); *FPC*—free phenol content (mg GAE/100 g). *BPC*—bound phenol content (mg GAE/100 g); *Ins Fiber*—insoluble fiber content (%); *Sol Fiber*—soluble fiber content (%); *TMA*—total monomer; anthocyanins (mg cyanidin-3-glucoside/100 g); *Free DPPH* (μmol TE/g); *Bound DPPH* (μmol TE/g); *Free RP*—free reducing power (μmol TE/g); *Bound RP*—bound reducing power (μmol TE/g); *Free ABTS*—free ABTS assays (μmol TE/g); *Bound ABTS*—bound ABTS assays (μmol TE/g).

**Table 3:** Response surface methodology results (Sum of squares).

	Chokeberry	Chokeberry^2^	Cinnamon	Ch × C	Error	*R* ^2^	adj. *R*^2^
df	1	1	1	1			
*SME*	17.01	329.88	134.32	2.29	581.76	0.54	0.33
*Torque*	5.12	89.12	8.77	0.55	304.05	0.30	0.00
*ER*	0.04	0.13	0.02	0.00	1.37	0.46	0.23
*BD*	0.00	0.00	0.00	0.00	0.00	0.08	0.00
*Hard*	54.62	12.08	43.96	27.14	244.22	0.42	0.17
*NoFract*	53.03	18.68	411.71	18.99	1080.01	0.57	0.37
*Crisp work*	0.05	0.00	0.03	0.00	4.96	0.20	0.00
*Crisp index*	0.02	0.01	0.02	0.01	0.06	0.55	0.35
L*	32.54	374.55 *	10.65	14.11	105.22	0.96	0.94
a*	0.51	11.54 *	0.42	0.61	5.94	0.91	0.87
b*	0.47	19.36 *	0.13	0.37	18.77	0.75	0.64
C*	0.04	2.88	0.00	0.01	12.04	0.26	0.00
*WAI*	0.01	1.63 *	0.04	0.26	2.16	0.74	0.62
*WSI*	1.47	8.77 *	2.21	3.51	11.96	0.63	0.47
*FPC*	595.51 *	218.10 *	235.72 *	181.16 *	294.50	0.99	0.99
*BPC*	154.30 *	0.05	118.54 *	20.94 *	27.83	1.00	0.99
*Ins Fiber*	5.36 *	1.27	0.00	0.45	3.10	0.98	0.98
*Sol Fiber*	0.20 *	0.28 *	0.35 *	0.26 *	0.22	0.73	0.61
*TMA*	38.33	2712.86	33.36	94.26	4953.92	0.42	0.16
*Free DPPH*	18.48 *	1.08	1.22	0.27	5.82	0.98	0.97
*Bound DPPH*	0.58 *	0.10 *	0.06	0.08	0.16	0.99	0.99
*Free RP*	5.25 *	0.15	0.55 *	0.38	0.95	0.98	0.98
*Bound RP*	0.00 *	0.00 *	0.00	0.00	0.00	0.97	0.96
*Free ABTS*	31.49 *	1.91	0.44	0.26	6.43	0.99	0.98
*Bound ABTS*	8.14 *	0.56	0.42	0.00	2.05	0.99	0.98

*R*^2^—coefficient of determination; adj. *R*^2^—adjusted coefficient of determination; *SME*—specific mechanical energy (Wh/kg); *Torque* (Nm); *ER*—expansion ratio; *BD*—bulk density (g/mL); *Hard*—extrudate hardness (kg); *NoFract*—crispness by number of fractures; *Crisp work*—crispiness work (Nmm); *Crisp index*—crispiness index (×10^−3^); L*—brightness from black to white; a*—(+/−) value indicates red/green chromaticity; b*—(+/−) value denotes yellow/blue chromaticity; C*—value assesses color intensity and saturation; *WAI*—water absorption index (g/g); *WSI*—water solubility index (g/100 g); *FPC*—free phenols content (mg GAE/100 g); *BPC*—bound phenol content (mg GAE/100 g); *Ins Fiber*—insoluble fiber content (%); *Sol Fiber*—soluble fiber content (%); *TMA*—total monomer. Anthocyanins (mg cyanidin-3-glucoside/100 g); *Free DPPH* (μmol TE/g); *Bound DPPH* (μmol TE/g); *Free RP*—free reducing power (μmol TE/g); *Bound RP*—bound reducing power (μmol TE/g); *Free ABTS*—free ABTS assays (μmol TE/g); *Bound ABTS*—bound ABTS assays (μmol TE/g). * significant at *p* < 0.05 level.

**Table 4:** Verification of RSM models.

Variable	χ^2^	RMSE	MPE	SSE	AARD	*R* ^2^
*SME*	44.751	6.446	3.500	581.762	3.500	0.733
*Torque*	23.388	4.660	3.144	304.048	3.144	0.552
*ER*	0.106	0.313	5.975	1.372	5.975	0.682
*BD*	0.000	0.006	8.791	0.000	8.791	0.289
*Hard*	18.786	4.177	5.737	244.222	5.737	0.650
*NoFract*	83.077	8.783	7.815	1080.006	7.815	0.752
*Crisp work*	0.381	0.595	12.447	4.957	12.447	0.445
*Crisp index*	0.005	0.068	12.704	0.064	12.704	0.743
L*	8.094	2.741	5.642	105.216	5.642	0.978
a*	0.457	0.651	5.348	5.937	5.348	0.953
b*	1.444	1.158	9.405	18.774	9.405	0.867
C*	0.926	0.927	5.018	12.036	5.018	0.509
*WAI*	0.166	0.393	5.984	2.160	5.984	0.859
*WSI*	0.920	0.924	2.289	11.956	2.289	0.797
*FPC*	22.654	4.586	4.419	294.503	4.419	0.995
*BPC*	2.141	1.410	2.696	27.831	2.696	0.998
*Ins Fiber*	0.238	0.470	2.271	3.096	2.271	0.992
*Sol Fiber*	0.017	0.125	42.224	0.218	42.224	0.855
*TMA*	381.070	18.811	288.647	4953.916	288.647	0.648
*Free DPPH*	0.448	0.645	5.539	5.825	5.539	0.991
*Bound DPPH*	0.012	0.106	4.498	0.156	4.498	0.997
*Free RP*	0.073	0.261	5.318	0.955	5.318	0.992
*Bound RP*	0.000	0.007	6.757	0.001	6.757	0.984
*Free ABTS*	0.494	0.678	5.841	6.426	5.841	0.994
*Bound ABTS*	0.158	0.383	10.619	2.052	10.619	0.995

χ^2^—chi square; RMSE—root mean square error; MPE—mean percentage error; SSE—sum of squared errors; AARD—average absolute relative deviation; *R*^2^—coefficient of determination; *SME*—specific mechanical energy (Wh/kg); *Torque* (Nm); *ER*—expansion ratio; *BD*—bulk density (g/mL); *Hard*—extrudate hardness (kg); *NoFract*—crispness by number of fractures; *Crisp work*—Crispiness work (Nmm); *Crisp index*—Crispiness index (×10^−3^); L*—brightness from black to white; a*—(+/−) value indicates red/green chromaticity; b*—(+/−) value denotes yellow/blue chromaticity; C*—value assesses color intensity and saturation; *WAI*—water absorption index (g/g); *WSI*—water solubility index (g/100 g); *FPC*—free phenol content (mg GAE/100 g); *BPC*—bound phenol content (mg GAE/100 g); *Ins Fiber*—insoluble fiber content (%); *Sol Fiber*—soluble fiber content (%); *TMA*—total monomer. Anthocyanins (mg cyanidin-3-glucoside/100 g); *Free DPPH* (μmol TE/g); *Bound DPPH* (μmol TE/g); *Free RP*—free reducing power (μmol TE/g); *Bound RP*—bound reducing power (μmol TE/g); *Free ABTS*—free ABTS assays (μmol TE/g); *Bound ABTS*—bound ABTS assays (μmol TE/g).

**Table 5:** Verification of ANN model.

	χ^2^	RMSE	MBE	MPE	SSE	AARD	*R* ^2^
*SME*	54.154	7.091	1.394	3.821	703.996	3.821	0.700
*Torque*	8.472	2.805	0.018	2.026	110.130	2.026	0.867
*ER*	0.023	0.146	−0.041	3.165	0.298	3.165	0.945
*BD*	0.000	0.004	−0.001	7.073	0.000	7.073	0.750
*Hard*	22.062	4.526	−1.394	5.673	286.809	5.673	0.626
*NoFract*	101.877	9.726	−1.374	8.141	1324.401	8.141	0.702
*Crisp work*	0.363	0.581	−0.042	11.882	4.723	11.882	0.497
*Crisp index*	0.006	0.076	0.012	11.578	0.081	11.578	0.671
L*	8.439	2.799	−0.498	4.784	109.706	4.784	0.978
a*	0.414	0.620	0.082	5.089	5.377	5.089	0.959
b*	0.808	0.866	0.048	7.088	10.502	7.088	0.928
C*	0.273	0.503	0.117	2.630	3.545	2.630	0.912
*WAI*	0.074	0.263	−0.021	4.261	0.968	4.261	0.941
*WSI*	1.519	1.188	0.397	2.777	19.747	2.777	0.696
*FPC*	115.092	10.338	−2.333	6.400	1496.197	6.400	0.987
*BPC*	4.770	2.105	0.139	5.691	62.008	5.691	0.996
*Ins Fiber*	0.683	0.796	−0.401	4.332	8.880	4.332	0.985
*Sol Fiber*	0.031	0.170	−0.002	53.782	0.403	53.782	0.709
*TMA*	879.883	28.584	14.376	69.860	11,438.479	69.860	0.109
*Free DPPH*	0.992	0.960	0.057	5.718	12.902	5.718	0.983
*Bound DPPH*	0.152	0.376	−0.137	10.279	1.979	10.279	0.974
*Free RP*	0.423	0.627	0.041	9.599	5.503	9.599	0.966
*Bound RP*	0.000	0.009	0.000	9.158	0.001	9.158	0.973
*Free ABTS*	3.771	1.871	−0.128	13.494	49.027	13.494	0.965
*Bound ABTS*	0.605	0.749	−0.190	17.860	7.860	17.860	0.983

χ^2^—chi square; RMSE—root mean square error; MBE—mean bias error; MPE—mean percentage error; SSE—sum of squared errors; AARD—average absolute relative deviation; *R*^2^—coefficient of determination; *SME*—specific mechanical energy (Wh/kg); *Torque* (Nm); *ER*—expansion ratio; *BD*—bulk density (g/mL); *Hard*—extrudate hardness (kg); *NoFract*—crispness by number of fractures; *Crisp work*—Crispiness work (Nmm); *Crisp index*—Crispiness index (×10^−3^; L*—brightness from black to white; a*—(+/−) value indicates red/green chromaticity; b*—(+/−) value denotes yellow/blue chromaticity; C*—value assesses color intensity and saturation; *WAI*—water absorption index (g/g); *WSI*—water solubility index (g/100 g); *FPC*—free phenol content (mg GAE/100 g); *BPC*—bound phenol content (mg GAE/100 g); *Ins Fiber*—insoluble fiber content (%); *Sol Fiber*—soluble fiber content (%); *TMA*—total monomer. Anthocyanins (mg cyanidin-3-glucoside/100 g); *Free DPPH* (μmol TE/g); *Bound DPPH* (μmol TE/g); *Free RP*—free reducing power (μmol TE/g); *Bound RP*—bound reducing power (μmol TE/g); *Free ABTS*—free ABTS assays (μmol TE/g); *Bound ABTS*—bound ABTS assays (μmol TE/g).

## Data Availability

The original contributions presented in the study are included in the article; further inquiries can be directed to the corresponding author.

## Abbreviations

The following abbreviations are used in this manuscript:

*T*	die temperature
*P*	pressure at the die
SME	specific mechanical energy
*ER*	expansion ratio
*BD*	bulk density
*Hard*	extrudate hardness
*NoFract*	crispness by number of fractures
*Crisp work*	crispiness work
*Crisp index*	crispiness index
*L**	brightness from black to white
*a**	value indicates red/green chromaticity
*b**	value denotes yellow/blue chromaticity
*C**	value assesses color intensity and saturation
*WSI*	water solubility index
*WAI*	water absorption index
*FPC*	free phenols content
*BPC*	bound phenols content
*Ins Fiber*	insoluble fiber content
*Sol Fiber*	soluble fiber content
*TMA*	total monomer
*Free RP*	free reducing power
*Bound RP*	bound reducing power
*Free ABTS*	free ABTS assays
*Bound ABTS*	bound ABTS assays
*χ*^2^	chi square
RMSE	root mean square error
MBE	mean bias error
MPE	mean percentage error
SSE	sum of squared errors
AARD	average absolute relative deviation
*R*^2^	coefficient of determination
